# Tandem mass tag-based proteomics analysis of type 2 diabetes mellitus with non-alcoholic fatty liver disease in mice treated with acupuncture

**DOI:** 10.1042/BSR20212248

**Published:** 2022-01-14

**Authors:** Guan Wang, Mengyuan Li, Shuo Yu, Mengqi Guan, Shiqi Ma, Zhen Zhong, Yihui Guo, Xiangyang Leng, Haipeng Huang

**Affiliations:** 1 College of Traditional Chinese Medicine, Changchun University of Chinese Medicine, Changchun 130117, China; 2 College of Acupuncture and Massage, Changchun University of Chinese Medicine, Changchun, 130117, China; 3 College of Integrated Traditional Chinese and Western Medicine, Changchun University of Chinese Medicine, Changchun 130117, China; 4 College of Traditional Chinese Medicine, Changchun University of Chinese Medicine, Changchun 130117, China

**Keywords:** glucose and lipid metabolism, hepatocyte steatosis, insulin resistance, nonalcoholic fatty liver disease, proteomics, type 2 diabetes mellitus

## Abstract

**Objective:** To explore the proteomics profiles of hepatocytes of mice treated with acupuncture for type 2 diabetes mellitus (T2DM) with non-alcoholic fatty liver disease (NAFLD). **Methods:** We used a Tandem mass tag (TMT)-based quantitative proteomics approach to identify proteins with potential molecular mechanisms associated with acupuncture interventions for T2DM with NAFLD. **Results**: Acupuncture effectively improved body weight, blood glucose, and insulin levels in T2DM with NAFLD mouse models and reversed steatosis within hepatocytes. Quantitative TMT-based proteomics analysis identified a total of 4710 quantifiable proteins and 1226 differentially expressed proteins (DEPs) in the model control group (MCG) compared with the normal control group (NCG). The Acupuncture Treatment Group (ATG) presented in 122 DEPs was compared with the MCG group. We performed a bioinformatics analysis, which revealed that DEPs enriched in the KEGG pathway after acupuncture treatment were mainly involved in the PPAR signaling pathway, fatty acid biosynthesis, fatty acid metabolism, fatty acid elongation, fat digestion and absorption. We used parallel reaction monitoring (PRM) technology to explore the association of aldehyde oxidase 1 (Aox1), acyl-coenzyme A thioesterase 2 (Acot2), perilipin-2 (Plin2), acetyl-CoA carboxylase 1 (Acc), NADP-dependent malic enzyme (Me1), fatty acid synthase (Fasn), ATP-citrate synthase (Acly), fatty acid-binding protein, intestinal (Fabp2) with lipid synthesis, fatty acid oxidation, and hepatocyte steatosis. **Conclusions:** Our results show that acupuncture can regulate the protein expression of T2DM in the NAFLD mice model, and can effectively improve hepatocyte steatosis, and has potential benefits for the clinical treatment of this disease.

## Introduction

Disorders in glycolipid metabolism are characterized by altered glucose and lipid metabolism, with neuroendocrine disorders, insulin resistance (IR), oxidative stress, altered inflammatory response, and intestinal flora imbalance as the main pathological manifestations, either alone or combined with changes in clinical blood glucose levels, dyslipidemia, non-alcoholic fatty liver disease (NAFLD), hypertension, and atherosclerosis [1]. This condition is not only a global disease, but also a serious health concern among Chinese individuals, worsening the quality of life of patients and placing a heavy medical and economic burden on society. According to the International Diabetes Federation (IDF), there were 463 million diabetes patients worldwide in 2019, representing 9.3% of the global population [2]; more than 90% of patients were diagnosed with type 2 diabetes mellitus (T2DM) [3,4], and of these approximately 70–75% of patients experienced NAFLD [5–7]. The two diseases often occur concomitantly, increasing not only the risk of diabetic complications, but also the risk of liver cirrhosis and liver cancer [8].

Studies have shown that abnormal glucose metabolism can lead to dyslipidemia. There are many mechanisms that cause T2DM with NAFLD, including IR, oxidative stress imbalance, accumulation of free fatty acids (FFAs), and endoplasmic reticulum stress (ERS). The reasons for these factors may be due to the activity of multiple signaling pathways, but these mechanisms need to be further clarified. Currently, modern medical treatment of T2DM with NAFLD is mainly based on strict diet control and pharmacological treatment with metformin [9], thiazolidinediones [10], α-glucosidase inhibitor [11], and the glucagon-like receptor agonist GLP-1 RA [12]. However, there is no specific drug for this disease and the clinical efficacy needs to be further confirmed. Several studies have shown that acupuncture has a beneficial effect on improving blood glucose and dyslipidemia in patients with disorders of glucose and lipid metabolism [13–16]. Electroacupuncture can reduce cholesterol levels and regulate liver gene expression in high-cholesterol mouse models [17]. Acupuncture has a positive regulatory effect on glucose and lipid metabolism, and its mechanism involves multiple signaling pathways and multiple targets. But there are few studies on the effects and mechanisms of acupuncture on liver lipid accumulation on diabetic status. The liver is the main organ of glucose and lipid metabolism, mechanisms closely related to the occurrence of T2DM with NAFLD. Thus, there is an urgent need to clarify the mechanism of acupuncture intervention in T2DM with NAFLD.

Proteins are not only the implementers of gene functions, but they are also the main bearers of life activities and play an obvious role in physiology. Quantitative proteomics is the accurate quantification and identification of all proteins expressed in a genome or all proteins in a complex mixed system [18]. Due to the advantages of proteomics technology, it has been widely used in the research field of Traditional Chinese Medicine (TCM) to explore bioregulatory mechanisms and has provided valuable information on biological mechanisms [19,20]. Isobaric tags for relative and absolute quantitation (iTRAQ) is an isobaric labeling method. It has often been used in acupuncture studies to identify biomarkers and to elucidate potential mechanisms for the treatment of diseases [21–23]. However, quantitative proteomics is seldom to study of the mechanism of acupuncture treatment in T2DM with NAFLD. In the present study, we used tandem mass tag-based (TMT) proteomics technology to reveal the potential mechanisms underlying acupuncture on T2DM with NAFLD. Our findings provide new insight and a theoretical basis for the clinical treatment of diseases of glucose and lipid metabolism by acupuncture.

## Materials and methods

### Animals and ethics statement

All animal experiments were performed at the animal experiment center of Changchun University of Chinese Medicine. Twenty-two db/db male mice (6 weeks old, weight 30 ± 3 g) and eleven db/m male mice (6 weeks old, weight 19 ± 2 g) were used in the study. Mice were obtained from the Peking University Medical Department (Beijing, China; License number: SCXK (Jing) 2016-0010). Food and water were freely available to mice, the temperature in the animal cages was controlled at 21 ± 1°C, with 40–70% relative humidity, and noise below 60 decibels. All mice were housed in cages under a reversed 12-h light/12-h dark cycle. The present study was approved by the Ethics Committee of the Changchun University of Chinese Medicine (Approval Number: 2021189). All experiments were performed in accordance with the guidelines for the nursing and use of experimental animals of Changchun University of Chinese Medicine.

### Animal treatment

All mice were housed adaptively for 7 days prior to the beginning of the experiment. Twenty-two db/db mice were randomly divided into acupuncture treatment group (ATG) and model control group (MCG), with 11 mice in each group, and 11 db/m mice reared under the same conditions were used as normal control group (NCG). At baseline, mice were measured for body weight, blood glucose and activity, and the results were used to remove outliers.

During the experiment, three groups of mice were given the same diet and water, and fixed at the same time, none of the NCG and MCG mice were excluded from the study. The ATG received electroacupuncture treatment. ATG therapy was administered once per day, for 20 min each time, and six treatments were counted as a course of treatment, with continuous treatment for two courses. The specific acupoints selected for acupuncture treatment included BL13 (Feishu), BL20 (Pishu), BL23 (Shenshu), LI4 (Hegu), ST36 (Zusanli), SP6 (Sanyinjiao), LR3 (Taichong), and bilateral acupoints were selected. Acupoints were located according to the atlas of acupuncture points ‘Experimental Acupuncture and Moxibustion’ (Beijing: China Traditional Chinese Medicine Publishing House Co., Ltd, 2016) [24]. All acupuncture operations were performed with 0.18*10-mm acupuncture needles (Hwato, Suzhou Medical Appliance Factory, Suzhou, China). The depth of BL13, BL20, and BL23 was 4 mm, ST36 was 3 mm, SP6 was 1.5 mm, LI4 and LR3 were 1 mm. The stimulation of acupoints was obtained using the Hwato SDZ-V electroacupuncture therapy instrument. BL13-BL23 and SP6-ST36 on the same side were selected as electroacupuncture connection points, the frequency of wave thinning was set at 3 Hz (frequency ratio 1:5), and the intensity was set so that the muscles of the stimulated parts could contract slightly and the mice could tolerate it. The treatment time was 20 min. Mice in NCG and ATG were only observed as controls.

During the course of the 2-week-treatment, body weight, food intake, and fasting blood glucose (FBG) were measured once per week. Before measuring FBG, the animals fasted for 12 h, and the tail-tip blood was used to obtain samples.

### Sample collection

After the measurement of basic indicators, mice were anesthetized by breathing isoflurane, and then liver samples were collected, and placed into the freezing tube, and snap-frozen in liquid nitrogen, and then stored at −80°C until use for proteomics and metabolomics analyses. Finally, mice were killed by cervical dislocation.

### Detection of serum fasting insulin by ELISA

Serum fasting insulin (FINS) was detected using the mouse FINS ELISA kit (Jiangsu Cote Biological Technology Co. Ltd., Product Number: KT2579). The homeostasis model assessment of IR (HOMA-IR) index was calculated according to the formula: (FBG value × FINS value)/22.5.

### Histopathological examination

Liver tissue fixed with paraformaldehyde was first immersed in an 80% alcohol solution overnight (>12 h), then dehydrated by sequential immersions in an alcohol gradient (90, 95, 100%) and xylene. The tissue was embedded in a wax block, sectioned, attached to a slide and dewaxed with xylene and graded alcohol. The tissue was stained with Hematoxylin and Eosin (H&E) (Sigma–Aldrich, Product Number: MHS16 and 318906), then dehydrated in a gradient alcohol solution as described above, and finally sealed with neutral gum. The morphological changes of the liver tissue were observed by light microscope.

### Oil Red O staining

Preparation of frozen sections occurred at the optimal cutting temperature (OCT) of embedded tissue, and liver tissue was cut into 8-μm sections. Accordingly, the staining protocol consisted of a 10-min fixation by 75% alcohol, and the tissue section was heated to 72°C for 2 h. Sections were soaked in distilled water for 10 min and immersed in the working solution of Oil Red O for 30 minutes. Thereafter, sections were differentiated in 60% isopropyl alcohol for 2 s and washed with distilled water. When desired, sections were counterstained with Mayer’s Hematoxylin for 4 min to visualize nuclei. The sections were then soaked in 1% ammonia for 5 s to return to blue and covered with a coverslip using 10% glycerol. Liver tissue sections were observed under light microscope.

### Liver tissue proteomics analysis

#### Protein extraction

Frozen liver tissues from mice in each group were collected and ground with liquid nitrogen to powder and then transferred to a 5-ml centrifuge tube. Subsequently, four volumes of lysis buffer (8 M urea, 1% Triton-100, 10 mM dithiothreitol, and 1% protease inhibitor cocktail) were added to the powder, followed by sonication three times on ice using a high-intensity ultrasonic processor (Scientz). After centrifugation at 12000×***g*** 4°C for 10 min, the supernatant was discarded. The protein was redissolved in 8 M urea and the protein concentration was determined with the BCA kit according to the manufacturer’s instructions.

#### Trypsin digestion

For digestion, the protein solution was reduced with 5 mM dithiothreitol for 30 min at 56°C and alkylated with 11 mM iodoacetamide for 15 min at room temperature in the dark. The protein sample was then diluted to urea concentration of less than 2 M. Finally, trypsin was added at a 1:50 trypsin-to-protein mass ratio for the first digestion overnight and 1:1000 trypsin-to-protein mass ratio for a second 4-h digestion.

#### TMT/iTRAQ labeling and HPLC fractionation

After trypsin digestion, the peptide was desalted using a Strata X C18 SPE column (Phenomenex) and vacuum dried. The peptide was reconstituted in 0.5 M TEAB and processed according to the manufacturer’s protocol for the TMT/iTRAQ kit. Briefly, one unit of TMT/iTRAQ reagent was thawed and reconstituted in acetonitrile. The peptide mixtures were then incubated for 2 h at room temperature, pooled, desalted, and dried by vacuum centrifugation. Tryptic peptides were fractionated into fractions by reverse phase HPLC with high pH using the Agilent 300Extend C18 column (5 μm particles, 4.6 mm ID, 250 mm length). Briefly, the peptides were first separated with a gradient of 8–32% acetonitrile (pH 9.0) over 60 min into 60 fractions. The peptides were then combined into 18 fractions and dried by vacuum centrifuging.

#### LC-MS/MS analysis

Tryptic peptides were dissolved in 0.1% formic acid (solvent A), directly loaded on to a homemade reversed-phase analytical column. The gradient consisted of an increase from 6 to 23% solvent B (0.1% formic acid in 98% acetonitrile) over 38 min, 23 to 35% in 14 min and climbing to 80% in 4 min, then holding at 80% for the last 4 min, all at a constant flow rate of 700 nl/min on an EASY-nLC 1000 UPLC system.

The peptides were subjected to NSI source followed by tandem mass spectrometry (MS/MS) in Q Exactive™ Plus (Thermo) coupled online to the UPLC. The applied electrospray voltage was 2.0 kV. The m/z scan range was 350–1000 for the full scan, and intact peptides were detected in the Orbitrap at a resolution of 35000. The peptides were then selected for MS/MS using the NCE setting as 27, and the fragments were detected in the Orbitrap at a resolution of 17500. A data-independent procedure alternated between 1 MS scan followed by 20 MS/MS scans. Automatic gain control (AGC) was set at 3E6 for full MS and 1E5 for MS/MS. The maximum IT was set at 20 ms for full MS and auto for MS/MS. The isolation window for MS/MS was set at 2.0 m/z.

#### Database search

Secondary mass spectral data were recovered using MaxQuant (v1.5.2.8). The database is SwissProt Mouse (16992 sequences), and the inverse library is added to calculate the false discovery rate (FDR) caused by random matching. Trypsin/P was specified as the cleavage enzyme allowing up to two missing cleavages, and the minimum length of the peptide was set as seven amino acid residues. The main search range was set to 5 ppm and 0.02 Da for fragment ions. The fixed modification was cysteine alkylation and the variable modification was methionine oxidation and N-terminal acetylation. The FDR was adjusted to <1% and the minimum score for peptides was set at >40.

#### Bioinformatics analysis

For hierarchical clustering based on different protein functional classifications (Gene Ontology [GO], KEGG Pathway), we first collated all categories obtained after enrichment along with their *P*-values and then filtered for those categories that were at least enriched in one of the clusters with *P*-value <0.05. This filtered *P*-value matrix was transformed by the function x = log_10_ (*P*-value). Finally, the x-values obtained were transformed to a z-score for each functional category. These z-scores were then clustered by hierarchical one-way clustering (Euclidean distance, average linkage clustering) in Genesis. The cluster membership was visualized using the ‘heatmap.2’ function from the R package ‘gplots’.

#### Differentially expressed protein quantification by parallel reaction monitoring

Parallel reaction monitoring (PRM) is based on high-resolution and high-precision mass spectrometry. It can selectively detect the target protein and the target peptides (such as the peptides modified after translation), so as to realize the absolute quantification of the target protein/peptide. Ten differentially expressed proteins (DEPs) were selected in the liver of db/db mice treated with acupuncture for PRM verification. The peptides were subjected to NSI source followed by MS/MS in Q Exactive™ Plus (Thermo) coupled online to the UPLC. The electrospray. Methods of protein extraction, trypsin digestion, LC parameters, electrospray voltage, scanning range, and Orbitrap resolution were performed using the same settings as the TMT experiment. followed by 20 MS/MS scans. AGC was set at 3E6 for full MS and 1E5 for MS/MS. The maximum IT was set at 20 ms for full MS and auto for MS/MS. The isolation window for MS/MS was set at 2.0 m/z. The resulting MS data were processed using Skyline (V.3.6). The quantitative information is normalized to the target peptide for relative quantitative analysis.

### Statistical analysis

All data were analyzed using Statistical Product and Service Solutions (SPSS) and graphs were prepared using GraphPad Prism 8 and expressed as mean ± SD. One-way analysis of variance (ANOVA) was used to calculate the statistical significance of the differences of the three groups. A *P*-value<0.05 means statistically significant.

## Results

### Acupuncture treatment alleviated T2DM with NAFLD-related indicators

**Figure 1:**
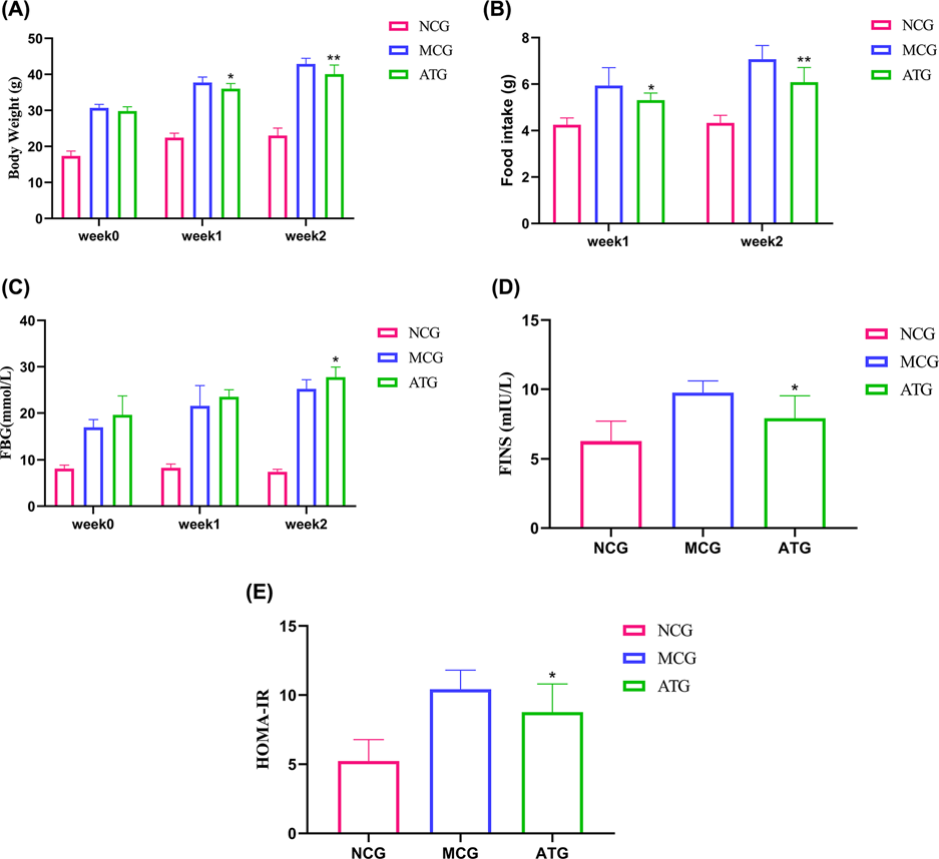
Effects of acupuncture treatment on general indicators of T2DM with NAFLD (**A**) Body weight and (**C**) changes in FBG before and after 1- and 2-week interventions. (**B**) Changes in food intake after 1- and 2-week interventions. (**D**) FINS was determined after treatment. (**E**) The HOMA-IR index was calculated according to the formula: HOMA-IR = (FINS × FBG)/22.5. Data were evaluated by ANOVA with LSD post hoc tests. *P<0.05, **P<0.01 vs MCG group. *n*=11 mice/group.

To evaluate the effects of acupuncture on T2DM with NAFLD mice, we measured body weight, food intake, FBG, and other disease-related indicators in each group. At baseline (week 0), the value of body weight and FBG of the mice in the MCG and ATG groups was significantly higher than in the NCG groups. After a 1-week-intervention, although FBG did not show significant differences between the MCG and ATG groups (*P*>0.05), body weight and food intake had changed significantly (*P*<0.05, Figure 1A,B). However, after a 2-week-intervention, the value of body weight (*P*<0.01, Figure 1A), food intake (*P*<0.01, Figure 1B), and FBG (*P*<0.05, Figure 1C) in the ATG group decreased significantly compared with the MCG group.

T2DM with NAFLD will alter insulin levels, reduce sensitivity to insulin, and eventually lead to IR. To show the effects of acupuncture treatment on insulin and evaluate the function of pancreatic islet β cells, we also calculated the HOMA-IR index based on FINS in mouse serum. The values of FINS and HOMA-IR showed significant differences in the MCG and ATG groups (*P*<0.05, Figure 1D,E). The results indicate that acupuncture treatment can improve IR, as evidenced by a reduction in the HOMA-IR index.

### Acupuncture treatment reduced hepatocyte steatosis in the db/db mouse model

**Figure 2:**
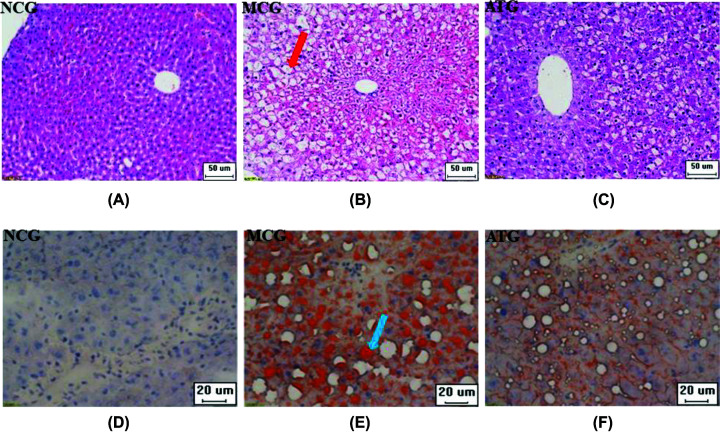
Effects of acupuncture treatment on hepatocyte steatosis of T2DM with NAFLD (**A–C**) H&E staining showing steatosis of hepatocytes, and a significant fat vacuole is indicated with the red arrow. (**D–F**) Oil Red O staining showing obvious lipid deposition in hepatocytes, and a significant lipid deposition is indicated by the blue arrow.

The morphological changes in the mouse liver were observed by H&E staining. As shown in Figure 2A–C, compared with the NCG group, the hepatocytes were disorganized, the hepatic lobules disappeared, and the hepatocytes were filled with large fat vacuoles, which pushed the nuclei to the subenvelope in the MCG. Acupuncture induced significant changes in the ATG group, compared with the MCG group: the fat vacuoles in the hepatocytes were smaller, distributed around the nucleus, and the hepatocytes were organized more regularly.

As shown in Figure 2D–F, the lipid deposition in hepatocytes was observed by Oil Red O staining. In the MCG group, a large amount of lipids stained bright orange by Oil Red O were deposited in the hepatocytes. After acupuncture treatment, the lipid deposition in hepatocytes was significantly lower than that in the MCG group and the steatosis of hepatocytes improved. There was no fat accumulation in the NCG group hepatocytes, thus only Hematoxylin-stained nuclei are shown.

### Proteomic profiling by TMT/iTRAQ analysis

#### Protein expression differences induced by acupuncture

**Figure 3:**
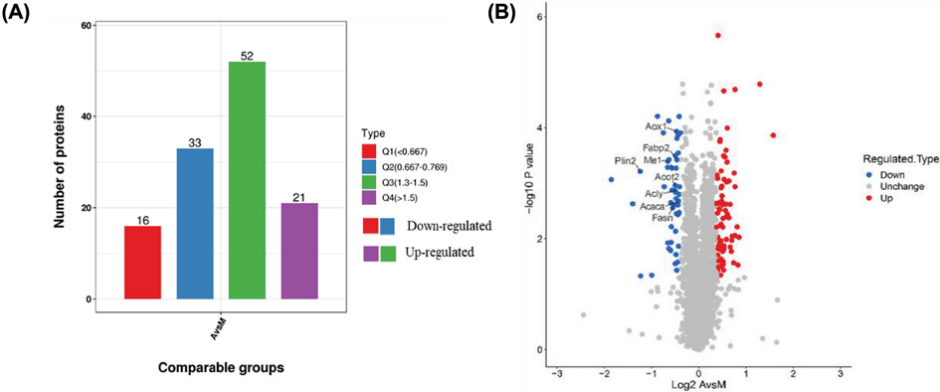
Quantitative analysis of proteins (**A**) The number of DEPs among A versus M, Q1 represents the A/M ratio < 0.667, Q2 represents the A/M ratio between 0.667 and 0.769, Q3 represents the A/M ratio between 1.3 and 1.5, Q4 represents the A/M ratio >1.5 (Q1 and Q2 are down-regulated proteins, and Q3 and Q4 are up-regulated proteins). (**B**) Volcano plot showing DEPs. The horizontal coordinate was the relative quantification of proteins after logarithmic transformation at the base of 2, and the vertical coordinate was the p-value of the test for the significance of differences after logarithmic transformation at the base of 10 (*n*=3 samples for each group).

In the present study, the differential expression of liver proteins in NCG, MCG, and ATG mice (three samples for each group) were identified and analyzed by TMT/ iTRAQ. Within the NCG, MCG and ATG groups, a total of 5242 proteins were identified. Among them, a total of 4710 proteins could be quantified. According to *P*-values <0.05, fold changes > 1.3 were considered significantly up-regulated, while differences <1/1.3 were considered significantly down-regulated proteins. A total of 122 DEPs (73 up-regulated and 49 down-regulated) were identified in the ATG group versus the MCG group. In total 1226 (381 up-regulated and 845 down-regulated) DEPs were identified between the NCG group and the MCG group (Table 1 and Figure 3A,B).

**Table 1:** A total of 122 DEPs regulated by acupuncture

Protein accession	Protein description	Gene name	A/M ratio	A/M P value	Regular Type
Q80T69	Lysine-specific demethylase 9	*Rsbn1*	2.983	0.000138	up
P46414	Cyclin-dependent kinase inhibitor 1B	*Cdkn1b*	2.446	0.0000165	up
P05366	Serum amyloid A-1 protein	*Saa1*	1.803	0.00944	up
P12246	Serum amyloid P-component	*Apcs*	1.776	0.0299	up
Q71KU9	Fibrinogen-like protein 1	*Fgl1*	1.755	0.00614	up
P04939	Major urinary protein 3	*Mup3*	1.701	0.00116	up
P97501	Dimethylaniline monooxygenase [N-oxide-forming] 3	*Fmo3*	1.701	0.00882	up
Q91W64	Cytochrome P450 2C70	*Cyp2c70*	1.701	0.0000206	up
Q01279	Epidermal growth factor receptor	*Egfr*	1.68	0.000659	up
O55003	BCL2/adenovirus E1B 19 kDa protein-interacting protein 3	*Bnip3*	1.667	0.0273	up
Q811D2	Ankyrin repeat domain-containing protein 26	*Ankrd26*	1.637	0.00898	up
P07361	α-1-acid glycoprotein 2	*Orm2*	1.622	0.0108	up
A2RSJ4	UHRF1-binding protein 1-like	*Uhrf1bp1l*	1.611	0.017	up
Q91V04	Translocating chain-associated membrane protein 1	*Tram1*	1.586	0.0142	up
Q8K182	Complement component C8 α chain	*C8a*	1.583	0.00242	up
Q45VN2	α-defensin 20	*Defa20*	1.555	0.00092	up
Q8CHG7	Rap guanine nucleotide exchange factor 2	*Rapgef2*	1.555	0.00422	up
Q61704	Inter-α-trypsin inhibitor heavy chain H3	*Itih3*	1.52	0.00364	up
Q6IR42	Zinc finger CW-type PWWP domain protein 1	*Zcwpw1*	1.518	0.000102	up
O88398	Advillin	*Avil*	1.514	0.000418	up
Q91Y47	Coagulation factor XI	*F11*	1.502	0.000955	up
P43025	Tetranectin	*Clec3b*	1.5	0.00408	up
Q91XL3	UDP-glucuronic acid decarboxylase 1	*Uxs1*	1.499	0.0103	up
Q64464	Cytochrome P450 3A13	*Cyp3a13*	1.491	0.000256	up
Q9JJH1	Ribonuclease 4	*Rnase4*	1.489	0.00239	up
P11609	Antigen-presenting glycoprotein CD1d1	*Cd1d1*	1.477	0.0031	up
Q8CJC7	Killer cell lectin-like receptor subfamily E member 1	*Klre1*	1.469	0.000341	up
Q923B6	Metalloreductase STEAP4	*Steap4*	1.449	0.000856	up
Q8K0C4	Lanosterol 14-α demethylase	*Cyp51a1*	1.446	0.0151	up
Q91YR9	Prostaglandin reductase 1	*Ptgr1*	1.444	0.0134	up
P01878	Ig alpha chain C region	—	1.443	0.0000218	up
Q8K2Y0	RING finger protein 219	*Rnf219*	1.438	0.000321	up
A6X935	Inter α-trypsin inhibitor, heavy chain 4	*Itih4*	1.436	0.0368	up
P56654	Cytochrome P450 2C37	*Cyp2c37*	1.427	0.0257	up
P11087	Collagen α-1(I) chain	*Col1a1*	1.426	0.0291	up
Q9D958	Signal peptidase complex subunit 1	*Spcs1*	1.425	0.00432	up
Q9JHI9	Solute carrier family 40 member 1	*Slc40a1*	1.42	0.017	up
Q9JLF6	Protein-glutamine γ-glutamyltransferase K	*Tgm1*	1.42	0.0062	up
Q61646	Haptoglobin	*Hp*	1.417	0.0291	up
Q9DBM0	ATP-binding cassette subfamily G member 8	*Abcg8*	1.411	0.00246	up
Q8C0L9	Glycerophosphocholine phosphodiesterase GPCPD1	*Gpcpd1*	1.405	0.0017	up
Q01149	Collagen α-2(I) chain	*Col1a2*	1.404	0.0151	up
Q8BZB2	Phosphopantothenoylcysteine decarboxylase	*Ppcdc*	1.404	0.0304	up
Q9QUQ5	Short transient receptor potential channel 4	*Trpc4*	1.403	0.00114	up
P31532	Serum amyloid A-4 protein	*Saa4*	1.4	0.00212	up
Q61490	CD166 antigen	*Alcam*	1.387	0.000602	up
P01027	Complement C3	*C3*	1.386	0.00122	up
Q8BH35	Complement component C8 β chain	*C8b*	1.386	0.011	up
Q05685	Folate receptor β	*Folr2*	1.384	0.0233	up
Q6XVG2	Cytochrome P450 2C54	*Cyp2c54*	1.384	0.00554	up
Q75N73	Zinc transporter ZIP14	*Slc39a14*	1.375	0.00578	up
P11589	Major urinary protein 2	*Mup2*	1.374	0.0453	up
Q04857	Collagen α-1(VI) chain	*Col6a1*	1.37	0.000165	up
P00186	Cytochrome P450 1A2	*Cyp1a2*	1.363	0.000178	up
Q05816	Fatty acid-binding protein 5	*Fabp5*	1.36	0.00222	up
Q9DB60	Prostamide/prostaglandin F synthase	*Fam213b*	1.358	0.003	up
P11714	Cytochrome P450 2D9	*Cyp2d9*	1.348	0.0131	up
P51658	Estradiol 17-β-dehydrogenase 2	*Hsd17b2*	1.346	0.0154	up
Q9QZZ6	Dermatopontin	*Dpt*	1.342	0.0251	up
Q9JJX6	P2X purinoceptor 4	*P2rx4*	1.339	0.0326	up
Q9JJ06	Glycoprotein-N-acetylgalactosamine 3-β-galactosyltransferase 1	*C1galt1*	1.333	0.0153	up
Q9QXC1	Fetuin-B	*Fetub*	1.326	2.18E-06	up
Q4U2R1	E3 ubiquitin-protein ligase HERC2	*Herc* *2*	1.325	0.0139	up
O70131	Ninjurin-1	*Ninj1*	1.322	0.00936	up
O88822	Lathosterol oxidase	*Sc5d*	1.322	0.0376	up
Q8CHQ9	N-acetyltransferase family 8 member 2	*Nat8f2*	1.322	0.00082	up
Q2VLH6	Scavenger receptor cysteine-rich type 1 protein M130	*Cd163*	1.32	0.0106	up
Q60847	Collagen α-1(XII) chain	*Col12a1*	1.32	0.0196	up
Q9R008	Mevalonate kinase	*Mvk*	1.32	0.00345	up
P51885	Lumican	*Lum*	1.31	0.000718	up
O89086	RNA-binding protein 3	*Rbm3*	1.306	0.00268	up
Q9JJ59	ATP-binding cassette subfamily B member 9	*Abcb9*	1.302	0.00616	up
Q921X9	Protein disulfide-isomerase A5	*Pdia5*	1.301	0.00232	up
Q9QZD8	Mitochondrial dicarboxylate carrier	*Slc25a10*	0.764	0.000124	down
Q8C5W3	Tubulin-specific chaperone cofactor E-like protein	*Tbcel*	0.753	0.00116	down
O55137	Acyl-coenzyme A thioesterase 1	*Acot1*	0.751	0.0016	down
P12791	Cytochrome P450 2B10	*Cyp2b10*	0.75	0.00338	down
Q9D379	Epoxide hydrolase 1	*Ephx1*	0.75	0.000063	down
Q5SWU9	Acetyl-CoA carboxylase 1	*Acaca*	0.743	0.0021	down
Q9WU66	Secreted frizzled-related protein 5	*Sfrp5*	0.742	0.0137	down
P48036	Annexin A5	*Anxa5*	0.74	0.000285	down
Q9DBE0	Cysteine sulfinic acid decarboxylase	*Csad*	0.737	0.0037	down
Q8BYR1	tRNA wybutosine-synthesizing protein 4	*Lcmt2*	0.736	0.00248	down
Q9DBL9	1-acylglycerol-3-phosphate O-acyltransferase ABHD5	*Abhd5*	0.736	0.000138	down
P07743	BPI fold-containing family A member 2 OS = *Mus musculus*	*Bpifa2*	0.732	0.0265	down
P52623	Uridine-cytidine kinase 1	*Uck1*	0.726	0.000157	down
P55050	Fatty acid-binding protein, intestinal	*Fabp2*	0.725	0.00038	down
Q9EQ21	Hepcidin	*Hamp*	0.723	0.0372	down
O54754	Aldehyde oxidase 1	*Aox1*	0.722	0.000117	down
P02089	Hemoglobin subunit β-2	*Hbb-b2*	0.719	0.00354	down
O35678	Monoglyceride lipase	*Mgll*	0.717	0.000537	down
Q9Z211	Peroxisomal membrane protein 11A	*Pex11a*	0.717	0.0019	down
P70279	Surfeit locus protein 6	*Surf6*	0.716	0.0194	down
P29758	Ornithine aminotransferase, mitochondrial	*Oat*	0.712	0.00146	down
A2AIL4	NADH dehydrogenase (ubiquinone) complex I, assembly factor 6	*Ndufaf6*	0.711	0.00736	down
P47934	Carnitine *O*-acetyltransferase	*Crat*	0.71	0.000319	down
E9Q309	Centrosome-associated protein 350	*Cep350*	0.708	0.0283	down
Q9QYR9	Acyl-coenzyme A thioesterase 2, mitochondrial	*Acot2*	0.708	0.0011	down
Q91V92	ATP-citrate synthase	*Acly*	0.705	0.00136	down
P19096	Fatty acid synthase	*Fasn*	0.698	0.00244	down
P97311	DNA replication licensing factor MCM6	*Mcm6*	0.682	0.00278	down
Q62452	UDP-glucuronosyltransferase 1-9	*Ugt1a9*	0.676	0.00134	down
P10648	Glutathione S-transferase A2	*Gsta2*	0.674	0.000537	down
P56655	Cytochrome P450 2C38	*Cyp2c38*	0.669	0.0061	down
Q8BUE4	Apoptosis-inducing factor 2	*Aifm2*	0.669	0.000524	down
P13516	Acyl-CoA desaturase 1	*Scd1*	0.667	0.0117	down
P19639	Glutathione S-transferase Mu 3	*Gstm3*	0.664	0.00224	down
O70571	[Pyruvate dehydrogenase (acetyl-transferring)] kinase isozyme 4, mitochondrial	*Pdk4*	0.662	0.0164	down
Q91WG1	Insulin-induced gene 2 protein	*Insig2*	0.646	0.000377	down
P29452	Caspase-1	*Casp1*	0.644	0.015	down
Q8BVZ1	Perilipin-5	*Plin5*	0.643	0.0000756	down
P06801	NADP-dependent malic enzyme	*Me1*	0.635	0.000404	down
P27786	Steroid 17-α-hydroxylase/17,20 lyase	*Cyp17a1*	0.634	0.0119	down
Q9D154	Leukocyte elastase inhibitor A	*Serpinb1a*	0.633	0.000519	down
Q62264	Thyroid hormone-inducible hepatic protein	*Thrsp*	0.602	0.00116	down
Q8VCH0	3-Ketoacyl-CoA thiolase B, peroxisomal	*Acaa1b*	0.594	0.000124	down
Q80W94	2-Acylglycerol* O*-acyltransferase 2	*Mogat2*	0.546	0.0000625	down
E9Q9R9	Disks large homolog 5	*Dlg5*	0.501	0.0455	down
Q8CCB4	Vacuolar protein sorting-associated protein 53 homolog	*Vps53*	0.426	0.0469	down
P43883	Perilipin-2	*Plin2*	0.423	0.000616	down
P16045	Galectin-1	*Lgals1*	0.379	0.00236	down
Q8BGT0	Osteopetrosis-associated transmembrane protein 1	*Ostm1*	0.277	0.000864	down

A, ATG; M, MCG. *n*=3 sample for each group.

#### Functional characterization of DEPs

**Figure 4:**
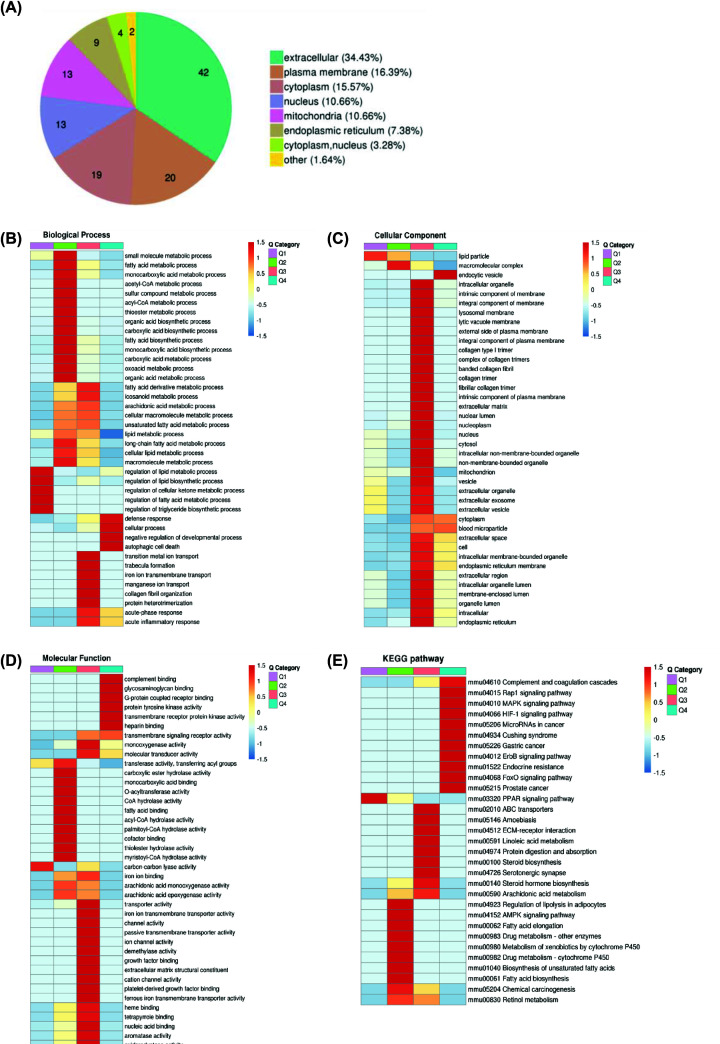
Functional enrichment analysis of DEPs in the liver after acupuncture treatment (**A**) Subcellular localization of DEPs. (**B–D**) The horizontal direction represents the enrichment test results of the different parts, and the vertical direction is the description of the differential expression enrichment related functions. Red indicates stronger degree of enrichment (the deeper the red color, the stronger the enrichment), and the blue color indicates a weaker enrichment (the lighter the blue, the weaker the enrichment). (**E**) Cluster analysis heatmap based on KEGG enrichment.

Classification of DEPs based on subcellular location. Most DEPs (*n*=42) were present in the extracellular space, and accounted for 34.43% of the whole; some proteins with significant changes in expression (*n*=20) were differentially expressed in the plasma membrane, which comprised 16.39% of the all DEPs; 15.57% of the significantly DEPs (*n*=19) were expressed in the cytoplasm; a large portion of the remaining DEPs were present in the nucleus, mitochondria, and endoplasmic reticulum (Figure 4A).

#### GO enrichment of differentially quantified proteins

DEPs was classified according to GO annotation into three categories: Biological process (BP), cellular component (CC), and molecular function (MF). Cluster analysis was carried out to compare the functional correlation of DEPs in the MCG and ATG groups, the GO enrichment analysis demonstrated that DEPs were associated with the single organism processes and cellular process in the BP (Figure 4B); organelle and cell in CC (Figure 4C); protein binding and catalytic activity in MF (Figure 4D). These results suggested that the improvements of T2DM with NAFLD associated with acupuncture treatment could be achieved by regulating glucose and lipid metabolism pathways.

To further explore the pathway of acupuncture-regulated liver glucose and lipid metabolism, we performed KEGG pathway enrichment analysis (Figure 4E). DEPs were mainly enriched in linoleic acid metabolism and were almost all up-regulated. The proteomics results indicated that acupuncture may affect changes in liver glucose and lipid metabolism by regulating related proteins in the linoleic acid synthesis and metabolism pathways.

### Validation of DEPs in liver by PRM

PRM is a modern tool of focused mass spectrometry and has been extensively used to quantify and define target proteins due to its specificity and sensitivity. Ten DEPs were validated by PRM in proteomics. As can be seen in Table 2, the results of proteomics verification in the liver of db/db mice showed that levels of aldehyde oxidase 1 (Aox1), acyl-coenzyme A thioesterase 2 (Acot2), perilipin-2 (Plin2), acetyl-CoA carboxylase 1 (Acc), NADP-dependent malic enzyme (Me1), fatty acid synthase (Fasn), ATP-citrate synthase (Acly), fatty acid binding protein, intestinal (Fabp2) in the MCG were 2.24, 4.58, 3.37, 4.09, 6.84, 7.71, 3.84, and 1.59 fold those of NCG, respectively. Combined with protein feature analysis, these eight proteins are associated with fatty acid synthesis and expression.

**Table 2:** PRM analysis of ten DEPs in the db/db mice liver

Protein accession	Protein	M/C ratio	M/C *P*-value	M/C ratio (TMT)	A/M ratio	A/M *P*-value	A/M ratio (TMT)
O54754	Aldehyde oxidase 1	2.24	4.58E-03	1.72	0.60	2.43E-03	0.72
Q9QYR9	Acyl-coenzyme A thioesterase 2	4.58	5.63E-04	2.91	0.79	7.55E-02	0.71
P43883	Perilipin-2	3.37	6.74E-02	2.42	0.29	3.28E-02	0.42
Q5SWU9	Acetyl-CoA carboxylase 1	4.09	1.53E-03	2.57	0.70	4.47E-02	0.74
P06801	NADP-dependent malic enzyme	6.84	1.26E-03	3.47	0.54	1.21E-02	0.64
P19096	Fatty acid synthase	7.71	1.89E-03	3.62	0.59	3.06E-02	0.70
Q91V92	ATP-citrate synthase	3.84	8.18E-04	2.46	0.60	7.38E-03	0.71
P55050	Fatty acid-binding protein 2	1.44	2.11E-01	1.59	0.76	3.02E-01	0.73
Q8VCH0	3-ketoacyl-CoA thiolase B,	4.83	7.74E-04	4.08	0.62	1.32E-02	0.59
P27786	Steroid 17-α-hydroxylase	3.34	5.16E-02	2.12	0.48	7.39E-02	0.63

A, ATG; C, NCG; M, MCG.

The PRM results confirmed that most of the ten DEPs exhibited comparable traits in abnormal glucose and lipid metabolism in the liver based on the TMT results, which supported the authenticity and reliability of the TMT data (Table 1). However, there are distinction effects between the TMT and PRM methods, which can potentially attributed to the differences in the specific detection techniques [25].

## Discussion

T2DM is an endocrine disease with high incidence, and patients are prone to metabolic syndromes such as NAFLD. The incidence of T2DM with NAFLD is more than twice that of healthy individuals [26]. The causes of T2DM in patients with NAFLD are related to dyslipidemia, IR, and genetic metabolic stress-induced liver injury. Difficulty in blood glucose control in T2DM with NAFLD will also increase, as they are reciprocal risk factors that form a vicious circle [27]. In this study, we established a mouse model of T2DM with NAFLD to investigate the pathophysiological mechanisms underlying glucose and lipid metabolism dysregulation. Obesity and abnormal glucose and lipid metabolism are typical symptoms of T2DM with NAFLD. Acupuncture reduced body weight, food intake, and FBG of T2DM with NAFLD mice (Figure 1A–C). Meanwhile, IR is also a determinant in T2DM with NAFLD. ATG can significantly reduce FINS and HOMA-IR levels of T2DM with NAFLD mouse models (Figure 1D,E), indicating that acupuncture achieves an improvement of IR. However, fat accumulation is also an important factor in T2DM with NAFLD. The results of the H&E staining and the Oil Red O staining showed that acupuncture could improve fat accumulation and inhibit steatosis in the liver of T2DM mice with NAFLD.

We used iTRAQ-based quantitative proteomics and metabolomics to comprehensively assess the changes in liver protein profiles of mice exposed to acupuncture. Different iTRAQ labeling reagents were used to label livers of T2DM with NAFLD animal model, and livers of control mice to detect significant changes in protein, and further bioinformatics analysis was performed to obtain the biological functions associated with the identified protein [28].

The GO annotation proteome was derived from the UniProt-GOA database, and provide a dynamically updated standardized vocabulary. We observed the enrichment of GO terms in the BPs of signal-organism, cellular, metabolic, and biological regulation in the acupuncture group. This indicated that acupuncture treatment of T2DM with NAFLD involves the regulation of glucose and lipid metabolism signal transduction, growth, and regeneration in mice. Furthermore, enrichment was observed in the annotations of the GO terms relating to MFs of binding, catalytic activity and transporter activity, implying that acupuncture can alter the levels of a large number of proteins in the T2DM with NAFLD mice liver that belong to organelles or cells and have binding and catalytic functions related to metabolic processes. Cellular composition GO analysis indicated enrichment in organelle, cell, membrane and extracellular region, which further indicated there was a DEP localized to in organelle and cell, during acupuncture treatment of T2DM with NAFLD mice. Additionally, enrichment was observed in organelles, cell membranes, and complicated mesh from structures composed of signaling polysaccharide macromolecules, which are important for glucose and lipid metabolism, and signal conduction. Therefore, cell membrane proteins play a vital role in the acupuncture treatment of T2DM with NAFLD. In addition, these effects indicated that the extracellular region is implicated in the initiation and development of T2DM with NAFLD.

In organisms, proteins perform a series of biochemical functions through interaction [29]. Pathway analysis is the most direct and basic method to understand the BP of disease-causing cells and to define potential mechanisms of intervention systematically and comprehensively. KEGG pathways analysis revealed that in the acupuncture treatment group, the top 20 enriched pathways involved fatty acid biosynthesis, biosynthesis of unsaturated fatty acids, chemical carcinogenesis, drug metabolism-cytochrome P450, metabolism of xenobiotics by cytochrome P450, the PPAR signaling pathway, fat digestion and absorption, regulation of lipolysis in adipocytes, fatty acid metabolism, fatty acid elongation, steroid hormone biosynthesis, retinol metabolism, drug metabolism-other enzymes, glycerolipid metabolism, pyruvate metabolism, arachidonic acid metabolism, glutathione metabolism, AMPK signaling pathway, peroxisome, and metabolic pathways. These pathways are related to glucose and lipid metabolism, indicating that acupuncture interferes with the process of liver glucose and lipid metabolism in T2DM mice with NAFLD. In conclusion, enrichment of the KEGG pathway identified pathways and signaling proteins are important for target selection in future research.

In our study, we selected eight of these proteins that are closely related to glucose and lipid metabolism for PRM validation: Aox1, Acot2, Plin2, Acc, Me1, Fasn, Acly, Fabp2. Aox1 is a highly expressed cytoplasmic enzyme in the liver and plays a key role in the metabolism of drugs containing aromatic heterocyclic substituents. Studies have shown that Aox1 generates reactive oxygen species that promote cell damage and fibrosis. Lipocalin reduces Aox1 expression by activating PPARα, and fatty liver is related to increased Aox1 expression [30]. The Acot family is widely distributed in tissues with high fatty acid oxidation capacity, such as liver, heart, and brown adipose tissue. Among them, Acot2 is distributed in mitochondria and can hydrolyze long-chain fatty acyl-CoA (LCFA-COA) in mitochondria to generate the corresponding LCFA and COA, promoting β-oxidation [31], and maintaining the balance function of acyl-CoA and CoA in mitochondria. Inside cells, lipids are stored in lipid droplets. Plin2 performs a key function in fatty acid absorption, lipid droplet formation, and lipid storage. Studies have shown that the increase in Plin2 has a negative effect on insulin sensitivity, leading to liver lipid accumulation [32,33]. Reduced Plin2 can prevent various metabolic disorders, such as obesity induced by a high-fat diet (HFD), IR, liver steatosis, adipose tissue inflammation, and T2DM [34–39]. The malic enzyme (Me) is an oxidoreductase, and NADP^+^ is the cytoplasmic form of the malic enzyme, which is expressed mainly in the liver and adipose tissue. Me1 is a key enzyme in endogenous fatty acid synthesis (Fas). The expression and activity of Me1 are closely related to the efficiency of Fas [40]. Fas, encoded by Fasn, catalyzes the precursor synthesis of saturated fatty acids. Fas involvement in the liver includes synthesis of lipids, which are stored as lipid droplets. Acc is a rate-limiting enzyme in Fas, which is activated by citric acid isomerism and inhibited by palmitic acyl coenzyme A. Acetyl coenzyme A and ATP inhibit isocitrate dehydrogenase and increase citric acid, thereby accelerating fatty acid synthesis. Therefore, Acc is closely related to the occurrence of this disease. Acly is a key enzyme linking glucose catabolism and lipogenesis, liver-specific knockout of Acly protects the liver from steatosis and dyslipidemia [41]. Fabp2 belongs to the family of fatty acid-binding proteins (Fabps). It is a group of cytoplasmic proteins that bind and transport long-chain FFAs. A mutation of the Fabp2 gene will change the structure and functional characteristics of Fabp2, thus affecting its affinity for long-chain fatty acids, which can lead to abnormal lipid metabolism [42].

In summary, Fasn plays an important role in the feeding behavior of mice, not only by controlling appetite and reducing body weight, but also by improving liver steatosis in mice; Acc, Me1, Fasn, and Acly play a role in the *de novo* synthesis of fatty acids in the liver; Aox1 and Acot2 promote β-oxidation of fatty acids; Plin2 and FABP2 are involved in lipid accumulation in hepatocytes. Our results indicate that the expression of Aox1, Acot2, Plin2, Acc, Me1, Fasn, Acly, Fabp2 in the liver of MCG mice was higher than that of NCG. After acupuncture treatment, the expression of these proteins decreased, indicating that acupuncture plays an interventional role at the beginning of fatty acid synthesis in the liver. By reducing the expression of Acc, Me1, Fasn, Acly, this leads to an inhibition of the synthesis of cholesterol and fatty acids, reducing body fat accumulation, and reduce body weight of T2DM with NAFLD mice, while inhibiting the expression of Aox1 and Acot2, promotes fatty acid β-oxidation. Meanwhile, lipid accumulation in hepatocytes was reduced by inhibiting the expression of Fabp2 and Plin2.

## Conclusions

Our data defined acupuncture as an effective and important treatment strategy for T2DM with NAFLD, and proved that acupuncture can reduce lipid storage in hepatocytes, inhibit *de novo* synthesis of fat, promote oxidation of fatty acids, and then reduce the risk of hepatocyte steatosis, by regulating the expression of Aox1, Acot2, Plin2, Acc, Me1, Fasn, Acly, and Fabp2. These findings provide further insights into the pathogenesis of glucose and lipid metabolism and the inhibition of these pathways may provide a rationale for acupuncture in the treatment of this disease.

## Data Availability

The data used to support the findings of this study are included within the article. Any further data can be made available from the corresponding author upon request.

## Abbreviations

A (ATG): acupuncture treatment group
Acc: acetyl-CoA carboxylase 1
Acly: ATP-citrate synthase
Acot2: acyl-coenzyme a thioesterase 2
AGC: automatic gain control
Aox1: aldehyde oxidase 1
BP: biological process
CC: cellular component
C (NCG): normal control group
DEP: differentially expressed protein
Fabp2: fatty acid-binding protein, intestinal
Fasn: fatty acid synthase
FBG: fasting blood glucose
FFA: free fatty acid
FINS: fasting insulin
GO: Gene Ontology
HOMA-IR: homeostasis model assessment of insulin resistance
H&E: Hematoxylin and Eosin
IR: insulin resistance
iTRAQ: isobaric tags for relative and absolute quantitation
KEGG: Kyoto Encyclopedia of Genes and Genomes
M (MCG): Model control group
Me1: NADP-dependent malic enzyme
MF: molecular function
MS/MS: tandem mass spectrometry
NAFLD: non-alcoholic fatty liver disease
Plin2: perilipin-2
PPAR: peroxisome proliferator-activated receptor
PRM: parallel reaction monitoring
TMT: tandem mass tag
T2DM: type 2 diabetes mellitus
